# Influence of leg length inequalities on pelvis and spine in patients with total hip arthroplasty

**DOI:** 10.1371/journal.pone.0221695

**Published:** 2019-08-27

**Authors:** Marcel Betsch, Roman Michalik, Maximilian Graber, Michael Wild, Rüdiger Krauspe, Christoph Zilkens

**Affiliations:** 1 Department of Orthopaedics, University Hospital RWTH Aachen, Aachen, Germany; 2 Department of Orthopaedics, University Hospital Duesseldorf, Duesseldorf, Germany; 3 Department of Orthopaedics, Traumatology and Hand Surgery, Klinikum Darmstadt, Darmstadt, Germany; Jagiellonian University Medical College, POLAND

## Abstract

**Background:**

Leg length inequalities (LLIs) are a common finding in patients with a total hip arthroplasty (THA). Therefore, we compared the effects of simulated LLIs in patients with total hip arthroplasty (THA) with a matched control group.

**Research question:**

Do LLIs lead to different effects on the musculoskeletal apparatus of patients with a THA then in a control group?

**Methods:**

In 99 patients with a THA the effects of simulated LLIs were compared to a matched control group of 101 subjects without a hip arthroplasty. First, we compared methods for LLI quantification (tape measurements, pelvic x- ray and rasterstereography). Second, the effects of simulated LLIs on the spine and pelvis were evaluated in both groups using surface topography. LLIs of 5, 10, 15, 20 and 30 mm were simulated on both sides with a simulation platform. The changes of pelvic position (pelvic obliquity & pelvic torsion) and the effects on spinal posture (surface rotation & lateral deviation) were measured and analysed using a surface topography system.

**Results:**

Mean LLI measured with a tape was 0.9 mm (SD +/- 14.8). Mean pelvic obliquity measured on x-rays was 1.2 mm (SD +/- 11.6) and with surface topography 0.9 mm (SD +/- 7.9). Simulated LLIs resulted in significant changes of pelvic position and spinal posture in the patient and control group. Interestingly, our study showed that simulated LLIs lead to greater changes in pelvic position (p<0.05) in patients with a THA.

**Significance:**

This is the first study to demonstrate that LLIs might have a greater impact on the pelvic position of THA patients than in native hips, which could indicate that LLIs do need to be compensated differently in patients with THA than in patients without a THA.

## Introduction

Leg length inequalities (LLIs) are present in up to 60–70% of the healthy population, and therefore they are a frequent finding in every orthopaedic clinic [1]. In patients with LLIs of 10mm and more up to 18.3% suffer from chronic back pain [2] and 30% of all patients with low back pain do have a LLI [3]. One of the most common causes for an acquired LLI in an adult population is total hip arthroplasty (THA). After a total hip replacement, LLIs of 3 to 17 mm can be found in 1 to 27% of all operated patients [4]. Problematic for such patients is that even minor LLIs can lead to clinical symptoms such as low back pain, sacro-iliac joint disorders or muscle imbalances [5, 6]. Edeen et al. found in their study that patients with LLI following THA were aware of the LLI in 32% of the cases, and that the majority of these patients were also clinically affected by the LLI [7]. In further studies, it was also shown that even minor LLIs of 9 mm after THA lead to an 18% worse Oxford Hip Score one year after surgery [8]. Finally, Wylde et al. found that five years after THA, one third of the patients that perceived a LLI had a significantly poorer functional outcome and reported a frequent limping [9]. However, the amount of pre- or postoperative LLIs that have a relevant clinical impact on the patient is still controversially discussed. This is mainly due to differences between the studies in measuring LLIs and differences in the documentation of clinical symptoms [1]. Major LLIs of 1.5 cm and more are known to cause gait disorders, lower back pain, greater trochanteric pain, aseptic prosthesis loosening or nerve irritation [10–12]. However, minor LLIs of 1 cm and less are usually well tolerated and compensated by the patients [13]. One of the reasons for conflicting reports on LLIs in patients undergoing THA is that different methods are used to measure LLIs. Currents standards in the measurement of LLI are tape measurements of leg length and determination of the level of the iliac crests in patients standing. However, both measurements have limited reliability and reproducibility compared to e.g. radiographic measurements [14–16]. The current gold-standard in determining LLIs are standing whole-leg anterior-posterior pelvic x-rays [10]. However, x-rays have the disadvantage of radiation exposure for patients and furthermore, they do not allow a functional assessment of the LLIs and their consequences. The exact biomechanical effects of LLIs on the hip joint, pelvis and spine remain uncertain using current standard measurement methods. A reason for conflicting reports on the influence of LLIs on patients might be the individual response of the patients´ spine and pelvis to LLIs [17]. Studies using surface topography have shown that kinematic chains exist in the human musculoskeletal system, which can be consequently affected by LLIs [18]. Surface topography is a method that allows to evaluate the effects of LLIs on the pelvic position and spinal posture, since it is a non-invasive imaging technique that uses light lines to scan and analyse the back surface of patients. This technique has shown its high validity and reliability in measuring LLIs and their effects on the spine and pelvis in numerous previous studies of patients without a THA [19– 21]. In previous studies, we were able to demonstrate a significant correlation between LLIs and changes in pelvic position and spinal posture [22]. Since LLIs following THA can lead to great patient dissatisfaction, we seek out to investigate and compare the effects of length inequalities in patients following THA with a control group of patients without an THA.

## Materials and methods

### Subjects

Ninety-nine patients with osteoarthritis of the hip (65 females and 34 males) following THA participated in this retrospective cohort study. Patients were measured on average 25 months (+/- 4.3) after total hip replacement surgery. THA was indicated due to severe clinical and radiographical signs of osteoarthritis of the hip. In all patients, a modular hip prosthesis was implanted (DePuy Synthes, Warsaw, IN, USA: S-Rom Modular Hip System and Pinnacle Acetabular cup). One surgeon with multiple years of surgical experience performed the THA. All patients were recruited during clinic visits for THA follow-up and were asked to participate in this study. A total of 101 matched healthy volunteers without a THA were included as a control group. There were no statistically significant differences between the two groups regarding age, height or weight (p = 0.178–0.319) (Table 1). All patients gave their oral and written consent to participate in this study. The independent ethics committee of the medical faculty of Duesseldorf University (Germany) approved the protocol of this study (Study Number: *3776*). Included were patients > 18 years after standard unilateral total hip replacement, who were able to stand on a simulation platform. Excluded were patients with non-standard THA due to e.g. hip dysplasia, Legg-Calvé-Perthes disease etc., as well as any prior history of fractures of the spine, pelvis and lower extremities. Patients with any knee or ankle joint replacements were also excluded.

**Table 1:** Epidemiological characteristics of the study group (left column) and control group (right column).

	THA group	Control group	p-value
*n*	99	101	
**Age (years)**	54.4 ± 13.4	52.6 ± 10.6	0.319
**Weight (kg)**	76.8 ± 16.6	73.9 ± 14.4	0.203
**Height (cm)**	168.7 ± 9.2	170.4 ± 9.1	0.178

## Methods

**Fig 1:**
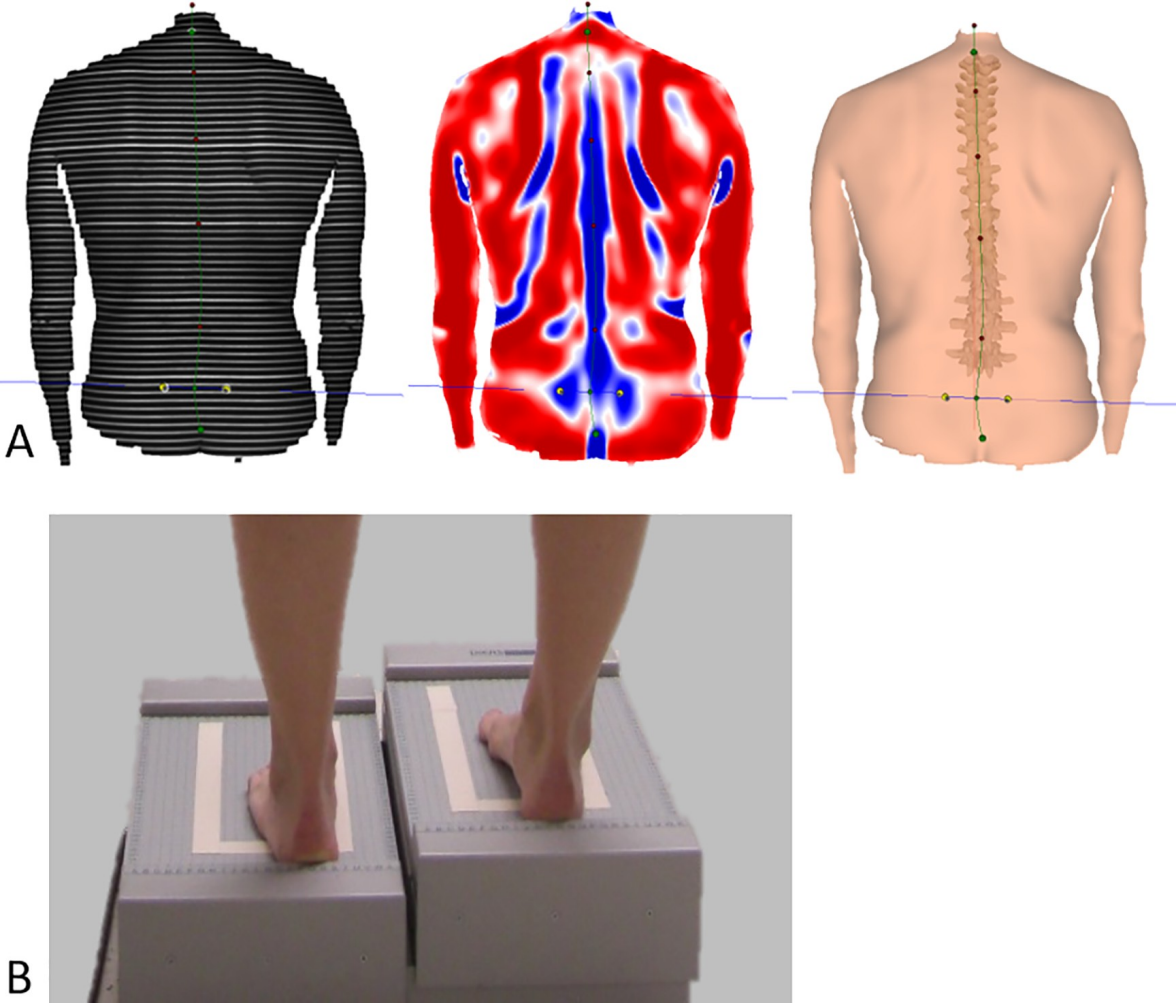
A) Rasterstereographic analysation of patients’ pelvic position and spinal posture. The raster is projected on the patients back, the deformation of the horizontal light lines and individual back shape is analysed to calculate a 3D-model of the spine and pelvis. B) Image of a patient standing on the simulation platform during measurements. The height of the platform can be controlled by a computer. All patients stood for 60 seconds on the platform to adapt to the simulated leg length inequality.

We used a surface topography system for the evaluation of the spine and pelvis to investigate for changes caused by the simulated LLIs (Formetric 4D, Diers International GmbH, Schlangenbad, Germany). We chose to use this method since it is widely distributed in Europe and foremost because it allows an immediate, radiation-free and three-dimensional analysis of the pelvic position and spinal posture. This system has been described in prior work [19, 22]. The technique of surface topography is based on Moiré topography [23] and was initially developed by Hierholzer and Drerup in the 1980s in Germany [24,25]. The device projects horizontal light lines onto the back surface of patients. A digital video camera records the deformation of the parallel light lines, which are caused by the patient specific anatomy (Fig 1A). Based on the recordings and a correlation model of the human spine, a 3D-model of the spine and pelvis can then be calculated [26]. The 3D-model is based on over 500 radiographs of the spine and pelvis for accurate reconstruction [27]. Studies show a good correlation of rasterstereographic and radiographic measurements for spinal and pelvic measurements [21], a high accuracy [20] and a high inter- and intrarater reliability, even under dynamic conditions [19, 28].

### Protocol

LLIs in patients that have undergone THA surgery were determined by tape measurements and by surface topography. For the tape measurements of LLI, we measured the distance between the anterior superior iliac spine (SIAS) and the tip of the medial malleolus in both legs (as previously described [1, 15]). LLI was then calculated as the difference in leg length between the two legs. Pelvic obliquity, as an indirect sign of LLI, was measured on standard anterior posterior (a.p.) pelvic x-rays. Therefore, patients were placed supine on an x-ray table with both legs hanging over the edge of the table to ensure reproducibility of the measurements. To determine pelvic obliquity, we drew a horizontal line through the ischial tuberosities. From this line, a perpendicular line was drawn to the tip of the greater trochanters. The distance in centimetres referred to the measured pelvic obliquity.

The assessment of LLI performed by surface topography was determined indirectly by measuring the position of the two lumbar dimples of the patients, which is expressed by the parameter pelvic obliquity. Pelvic obliquity is defined as the amount of tilt (measured in millimetres) from a horizontal line between two lumbar dimples [29]. The exact position of the lumbar dimples (DL and DR) was assessed by surface topography and used to calculate pelvic obliquity regarding their close relation to posterior superior iliac spines. In case of a positive value the right dimple (DR) is higher than the left dimple (DL), and in case of a negative value the DL is higher than DR. This technique also allows to measure pelvic torsion, which is defined as the degree of rotation of DL and DR to each other [26, 29].

In this study, we simulated LLIs, in order to examine their effects on pelvic position and spinal posture between patients with THA and a control group. Subjects were placed on a stand platform, whose height could be precisely controlled and which has been used in previous studies (Fig 1B) [22]. The weight distribution between both legs was measured by the platform to ensure an equal weight distribution between both legs. All patients and volunteers of the control group stood for 60 seconds on the platform prior to the measurements to adapt to the simulated LLIs. Patients were instructed to stand in a relaxed posture on the platform with extended knees and arms hanging on the sides of the torso, which is considered the neutral standing position. The following LLIs were simulated with the stand platform: 0 mm (neutral standing position), right (+) and left (-) leg LLIs of 5, 10, 15, 20 and 30 mm. Two trials of measurements with each of the respective simulated LLIs were performed and the mean value of each of these measurements was used for further statistical analysis.

### Data analysis

**Fig 2:**
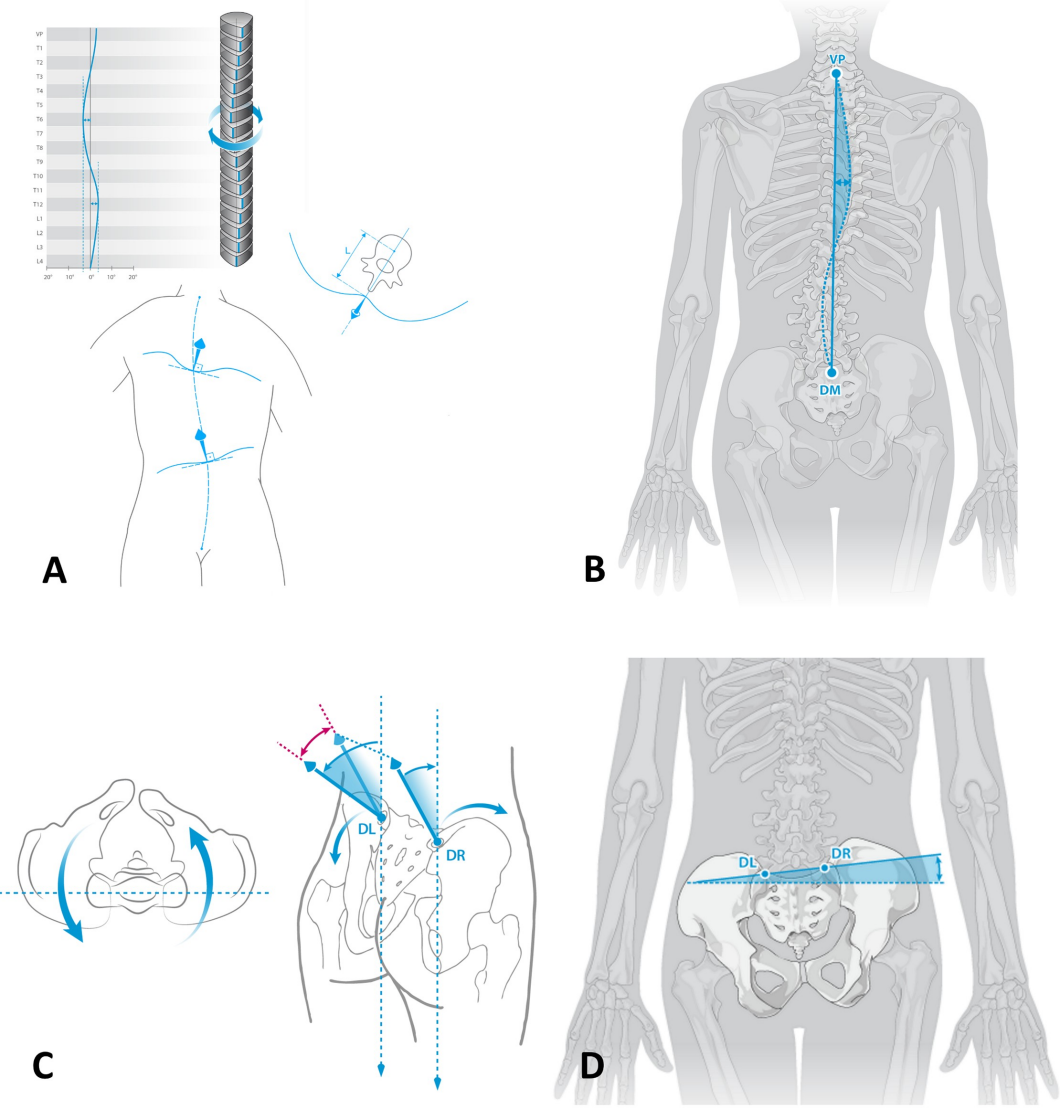
Surface rotation (A) of the spine is defined as the value of the horizontal components of the surface normals on the line connecting the spinous processes of the spine. Lateral deviation of the spine (B) is the deviation of its midline from a virtual line between vertebra prominence (C7) to the midpoint between left and right lumbar dimple. Pelvic torsion (C) is the torsion of the surface normals on the two lumbar dimples. Pelvic obliquity (D) is considered as the different height of the two lumbar dimples to each other.

We chose to measure and compare two pelvic and two spinal parameters that were most affected by the simulated LLIs in prior studies [22,30]. For the purpose of this study it is therefore necessary to define certain terms regarding the parameters that were measured (Fig 2): Surface rotation of the spine is measured in degrees (Fig 2A). It is defined as the value of the horizontal components of the surface normals on the line connecting the spinous processes of the spine. Lateral deviation (Fig 2B) of the spine is defined as the deviation of its midline from a virtual line between vertebra prominence (C7) to the midpoint between left and right lumbar dimple. Pelvic obliquity (as defined above) is the tilt (measured in millimetre) from the horizontal line between the lumbar dimples (Fig 2C). Pelvic torsion (Fig 2D) is the rotation of the lumbar dimples (DL and DR). It is measured in degrees. A positive pelvic torsion indicates a further anterior localization of the right lumbar dimple.

### Statistical analysis

Statistical analysis was performed with use of SPSS statistics software Version 22.0 (SPSS Inc., Chicago, USA). All data were checked for Gaussian distribution by the Kolmogorow-Smirnow-test and presented as means with standard deviations or 95% confidence intervals. Student t-Tests were used to determine differences between the groups. The level of significance was set at p < 0.05. Pearson correlation coefficient was performed between measurement methods.

## Results

First, we determined and compared LLIs or pelvic obliquity measured by three different established methods as previously described (Table 2). Mean LLI and pelvic obliquity following THA ranged from 0.9 to 1.2 mm measured with three different methods.

**Table 2:** Comparison of different measurement methods assessing LLIs or pelvic obliquity in ninety-nine patients after receiving a THA at our institution.

	Tape measurements	X- Ray	Surface topography
**Mean (mm)**	0.9	1.2	0.9
**SD**	14.8	11.6	7.9

LLIs measured with measurement tape showed a significant, however weak correlation with rasterstereographic (r = 0.304; p = 0.004) and X-ray based measurements (r = 0.285; p = 0.032) of pelvic obliquity. Pelvic obliquity measured by rasterstereography showed the highest correlation with radiographs (r = 0.494; p<0.001) as shown in Table 3.

**Table 3:** Pearson´s correlation between the three methods and standard deviations as shown. Significance level was set at p<0.05.

	Tape measurements	X- Ray	Surface topography
**Tape measurements**	1	R = 0.285 p-value = 0.032	R = 0.304 p-value = 0.004
**X- Ray**	R = 0.285 p-value = 0.032	1	R = 0.494 p-value< 0.001
**Surface topography**	R = 0.304 p-value = 0.004	R = 0.494 p-value< 0.001	1

A comparison of the spine and pelvic parameters between the operated and non-operated group in the neutral standing position without any simulated LLIs, revealed statistically significant differences between the two groups only for the parameter pelvic torsion (right side: p = 0.023 left side: p = 0.039) (Table 4).

**Table 4:** Comparison of spine and pelvic parameters between the two groups in the neutral standing position without any simulated LLIs.

Simulated LLI	0mm THA left		0mm THA right	
Group	Control	Patient	Control	Patient
Pelvic torsion (degr.)	-0.13 ± 2.42	1.00 ± 4.10	-0.13 ± 2.42	-1.40 ± 3.25
	(p = 0.039)		(p = 0.023)	
Surface rotation (degr.)	3.99 ± 1.69	4.50 ± 1.68	3.99 ± 1.69	4.46 ± 1.55
	(p = 0.097)		(p = 0.174)	
Lateral deviation (mm)	5.68 ± 3.34	5.93 ± 3.03	5.68 ± 3.34	5.07 ± 1.98
	(p = 0.665)		(p = 0.341)	
Pelvic obliquity (mm)	-0.30 ± 5.25	0.14 ± 6.71	-0.30 ± 5.25	-0.98 ± 6.59
	(p = 0.669)		(p = 0.560)	

**Fig 3:**
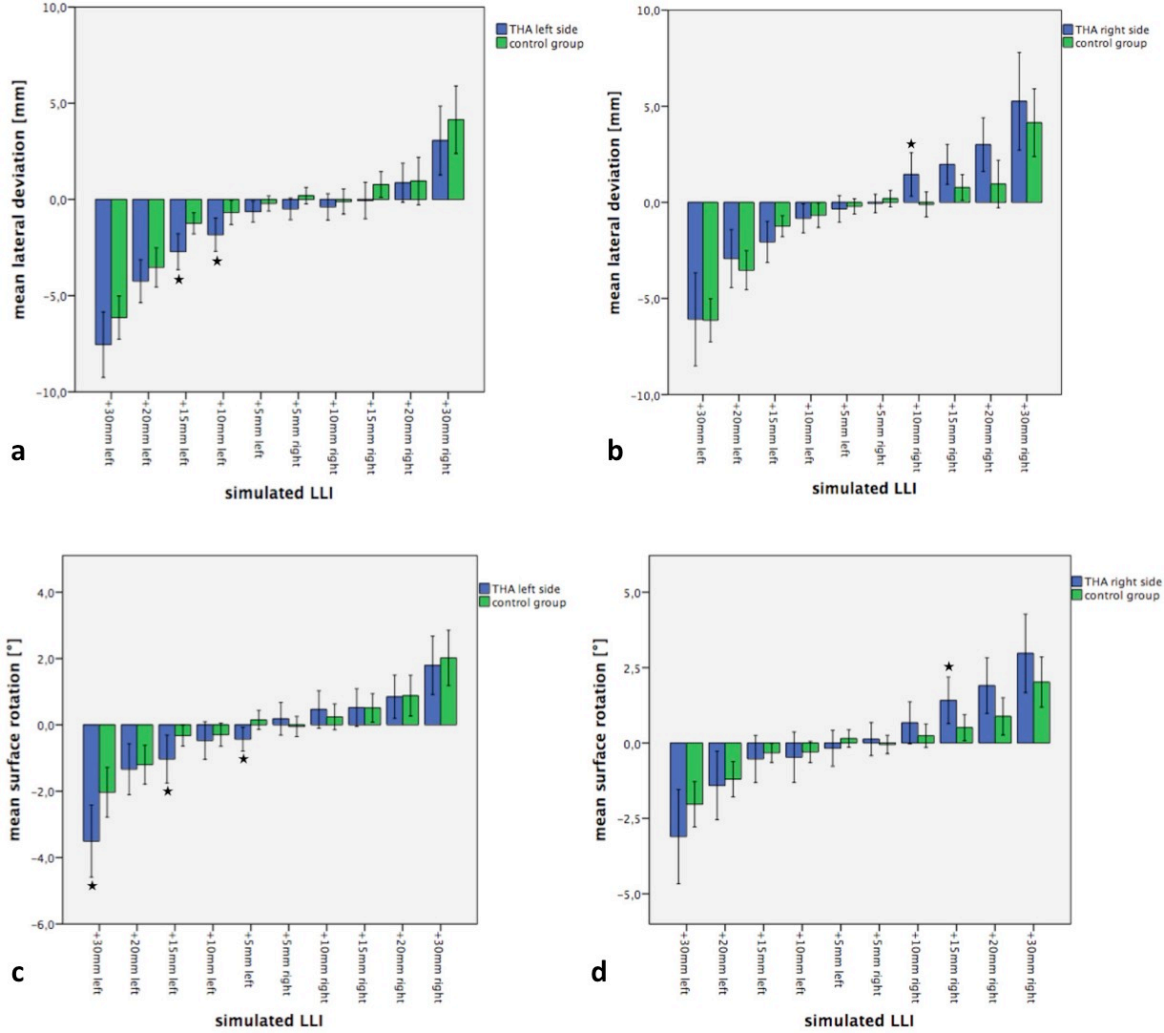
Effects of simulated LLIs on pelvic position of patients with and without THA. a) Changes of pelvic obliquity. Imaged for patients after THA of the left side (blue bar columns) and control group of healthy subjects (green bar columns). LLIs were simulated on the operated left side and the non-operated right side. b) Results for patients after THA of the right side with corresponding control group. LLIs were simulated on the leg of the THA and the contralateral leg. c) Changes of pelvic torsion in patients after THA of the left side and the control group of healthy subjects. LLIs were simulated on the operated left side and the contralateral side. d) Results for patients after THA of the right side with corresponding control group. LLIs were simulated on the leg of the THA and the contralateral leg. Significant differences (p<0.05) are marked with a star.

Analysis showed several statistically significant differences between the THA and the control group for the pelvic position and spinal posture in response to the simulated LLI. Changing the platform height resulted in a significant increase in pelvic obliquity in the control and in the patient group (THA). The THA patients’ pelvic obliquity was affected more by the simulated LLIs than the control group (Fig 3A and 3B).

For patients with a THA on the left side, the results showed a significant higher pelvic obliquity for simulated LLIs of 20 mm (p = 0.032) and 30 mm (p = 0.031) compared to the control group without surgery (Fig 3A). In patients with a left THA, simulated LLIs less than 20 mm did not result in significant differences between the two groups (p>0.05). Patients with a THA on the right side showed statistically greater pelvic obliquity, as compared to the control group, for LLIs of 10mm (p = 0.041), 20mm (p = 0.042) and 30mm (p = 0.029) (Fig 3B). Pelvic torsion also changed significantly with increasing LLIs on the ipsilateral side (Fig 3C and 3D). Pelvic torsion was significantly higher in patients with a THA on the left side for simulated LLIs of 10mm (p = 0.009) and 15mm (p = 0.005), as compared to the control group. All other heights showed a trend for higher values in the THA group, compared to the control group, without statistical significance (p>0.05).

**Fig 4:**
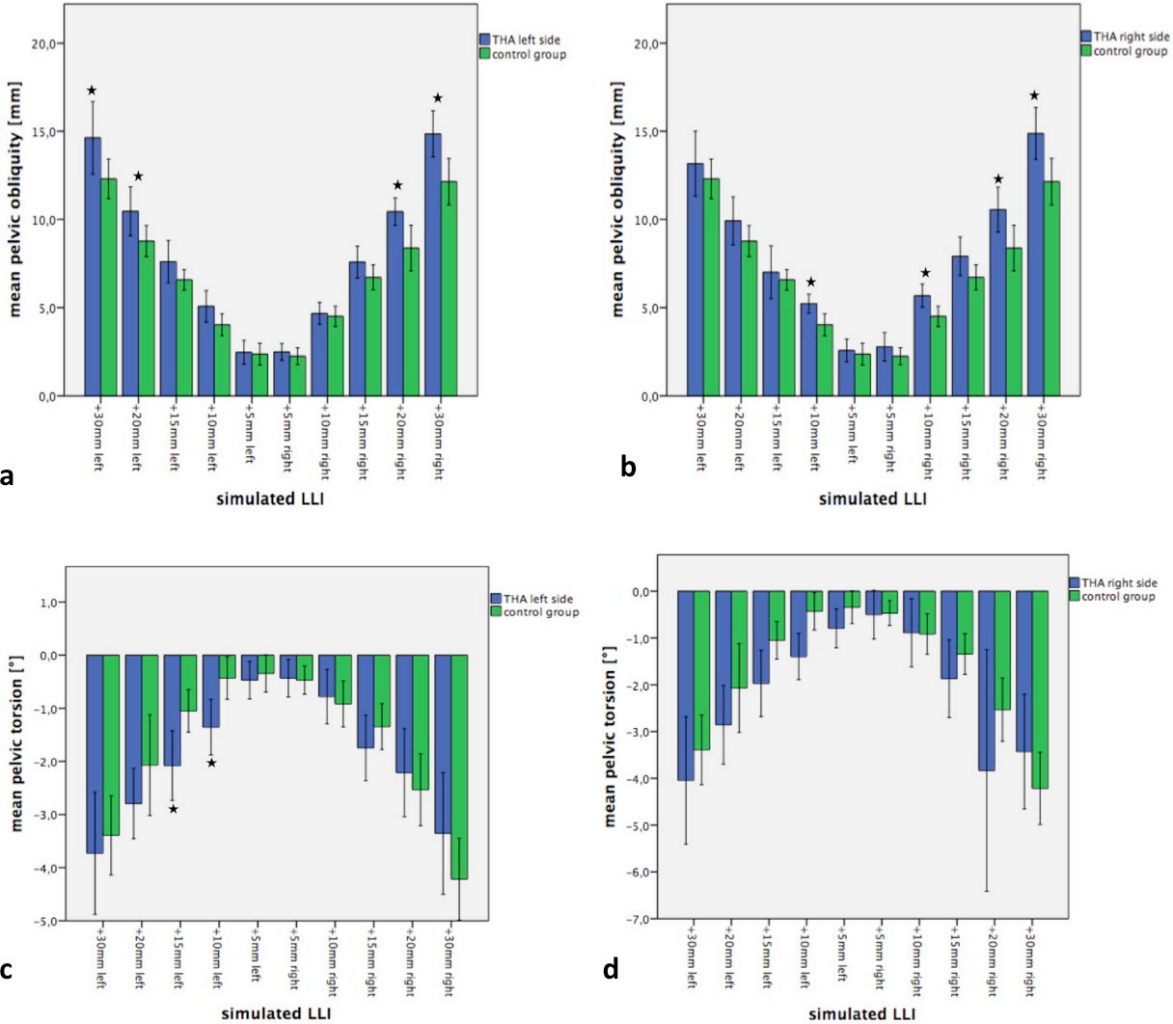
Effects of simulated LLIs on spinal posture of patients with and without THA. a) Changes of lateral deviation in patients after THA of the left side and the control group of healthy subjects. LLIs were simulated on the operated left side and the contralateral side. b) Results for patients after THA of the right side with a corresponding control group. LLIs were simulated on the leg of the THR and the contralateral leg. c) Changes of surface rotation after THA of the left side and control group of healthy subjects. LLIs were simulated on the operated left side and the contralateral side. d) Results for patients after THA of the right side with corresponding control group. LLIs were simulated on the leg of the THR and the contralateral leg. Significant differences are marked with a star.

Regarding spinal posture, the simulated leg length differences led to a significant increase of surface rotation and lateral deviation of the spine (Fig 4). The lateral deviation in patients with a THA on the left side was more affected by the simulated LLIs as compared to the control group for LLIs of 10mm (p = 0.039), and 15mm (p = 0.004) and for patients with a THA on the right side for LLIs of 10mm (p = 0.024) (Fig 4A and 4B). For all other simulated LLIs, we found a trend for a greater change of the lateral deviation to the simulated LLI as compared to the control group (p>0.05). Interestingly, no differences were found between the two groups when LLIs were simulated on the non-operated leg (p>0.05). The surface rotation describes the rotation of the spinous processes in degrees, which can be influenced by the simulated LLIs. Compared to the previous parameters, the surface rotation also significantly increased with increasing LLIs (Fig 4C and 4D). We found higher values of surface rotation in patients with a THA as compared to the control group, only on the operated side. These findings were statistically significant for simulated LLIs of 5mm (p = 0.013), 15mm (p = 0.044) and 30mm (p = 0.024) mm on the operated left leg and for an LLI of 15mm (p = 0.032) of the operated right leg (Fig 4C and 4D).

## Discussion

The purpose of this present study was to evaluate if patients with a THA show a different response to LLIs than subjects without a THA. This has some clinical importance since LLIs after THA are quite common and often do need to be treated. In previous studies examining non-operated patients, we were able to demonstrate that simulated LLIs can cause significant changes in pelvic position and spinal posture [19, 30]. The results of this current study confirm these findings that even in patients with a THA, LLIs have a significant influence on pelvic obliquity, pelvic torsion, lateral deviation and surface rotation of the spine. Following THA, we found a mean LLI of 0.9 mm using tape measurements, surface topography and 1.2 mm using x-rays. In the current literature, LLIs post THA range between 3 to 17 mm [4]. Other studies that assessed similar sized patient groups found mean LLIs after THA between 9.7 mm [7] and 15.9 mm [31]. A possible explanation for the smaller LLIs found in our study could be the difference in methods used to measure LLIs [15, 16]. A further explanation for the smaller mean LLI in our cohort could be differences between studies e.g. in the choice of anaesthesia [32], the use of cement [33], different implanting strategies [34]. In this present study, we also used a modular hip system, which seems to allow a more accurate restoration of leg length compared to other systems. As we analysed three different clinical methods for LLI measurement, we found a decent correlation between the used methods. Tape measurements show a poor correlation with rasterstereography due to the differences in the landmarks used to quantify LLIs. While tape measurements are based on a direct measurement of leg length, e.g. from the superior anterior iliac spines to the tip of the medial malleolus, the rasterstereographic method and X-ray indirectly measure LLIs. It therefore doesn’t surprise that the highest correlation was shown between both indirect LLI measurements by rasterstereography and X-ray.

Main purpose of this study was to assess the response of pelvis and spine to simulated LLIs in patients with a THA compared to a control group. In the current clinical practice, THA associated LLIs are treated like any other functional or anatomical leg length discrepancy. Regarding the proposed changes in biomechanics caused by a THA, such as e.g. tissue tension and stiffness, we hypothesised that LLIs after THA affect the pelvis and spine differently than in subjects with non-replaced hip joints. Our results confirm that the pelvis is significantly more affected by simulated LLIs in patients with a THA than in a matched control group. Simulated LLIs lead to an increase in pelvic obliquity and pelvic torsion in both the control and THA group. However, simulated LLIs in THA patients lead to even greater changes of the pelvic position compared to a matched control group, as shown by our results. Similar, however statistically non-significant findings were observed for surface rotation and lateral deviation of the spine. Previous studies have shown that there do exist kinematic chains in the musculoskeletal system that affect each other [22]. Studies that addressed these kinematic chains and the effects of THA on the spine, showed that the outcome after THA is highly influenced by pre-existing lumbar spine disorders [35] and that a THA can have a positive effect on these disorders like low back pain [36]. THA may not only lead to biomechanical changes of the hip joint itself, but also to changes in muscle balance, tendon and soft tissue stiffness, which all seem to reduce the ability of the musculoskeletal apparatus to adapt to LLIs. Known consequences of LLIs are compensatory lumbar scoliosis and lower back pain [2], a decrease of the centre-edge angle of the longer leg, potentially leading to premature osteoarthritis of the hip [37] and overall changes in gait [38]. Our results indicate that due to the biomechanical changes of the artificial hip joint and potentially due to soft tissue changes caused by the surgery and the THA itself, simulated LLIs may affect the pelvic position and spinal posture to a greater extent than in patients without a THA. Furthermore, our results demonstrate that these greater effects in THA patients are mostly found on the operated leg and not on the non-operated side.

Goals of THA procedure are to restore femoral offset, leg length and the center of hip rotation to reconstruct the physiological biomechanics of the hip joint. A qualitative analysis and comparison of femoral offset in postoperative X-ray of our patients showed no major differences. However, we did not measure and quantify femoral offset in this study, but it would be of clinical interest to do so in future studies.

A limitation of our study is that we evaluated acute and simulated LLIs and their effects on the pelvis and spine, instead of long-standing anatomical LLIs. However, we chose to use this previously used model to check if there were any differences at all between patients with and without THA. Further studies are necessary to evaluate, if the here found differences can also be found in THA patients with “real” greater LLIs. Another limitation of this present work is that LLIs were only examined under static conditions, while the subjects were standing on a simulation platform. We believe that additional studies in patients with LLIs under dynamic conditions would be of great interest. However, it must be noted that the clinical diagnosis and treatment of LLIs still often takes place in a standing, static position with extended knees. The rasterstereographic evaluation of pelvic obliquity provides a similar but more observer independent method. The tape-measurement as a direct evaluation of leg-length shows similar results in our study but limited relation due to its different methodology. We also chose not to clinically evaluate our patients and the effects of LLI in our patient cohort, because we believe that the here found LLIs of < 1 cm have not negatively affected our patients.

All patients were clinically examined for signs of lumbar spine degeneration. However, no x-rays were performed and therefore in future studies, it would be of interest to perform x-rays of the lumbar spine to be able to quantify lumbar spine degeneration.

In conclusion, this study shows that surface topography can be used to measure pelvic obliquity in patients with THA. The measured LLIs are similar to the obtained tape and radiographic measurements. In addition, this is the first study to demonstrate that LLIs might have a greater impact on the pelvic position of THA patients than in native hips, which could indicate that LLIs do need to be compensated differently in patients with THA than in patients without a THA.

## Supporting information

S1 Table Data underlying rasterstereographic measurements on patients and control group. (DOCX)
