# Supplementary material for: Influence of leg length inequalities on pelvis and spine in patients with total hip arthroplasty
Source: PLoS One. 2019 Aug 27;14(8):e0221695. doi: 10.1371/journal.pone.0221695 (PMC6711516; doi:10.1371/journal.pone.0221695)
Supplement: S1 Table — (DOCX) [file pone.0221695.s001.docx]

Table 1: Data underlying rasterstereographic measurements on patients and control group.

| THA right side and control group. LLI simulation on the right side | | | | | | | | | | | |
| --- | --- | --- | --- | --- | --- | --- | --- | --- | --- | --- | --- |
| Parameter | Group | 5mm | | 10mm | | 15mm | | 20mm | | 30mm | |
|  |  | Mean | SD | Mean | SD | Mean | SD | Mean | SD | Mean | SD |
| Pelvic obliquity [mm] | THA | 2.78 | 2.11 | 5.68 | 1.72 | 7.91 | 2.87 | 10.55 | 3.36 | 14.87 | 3.23 |
|  | Control | 4.51 | 2.91 | 6.72 | 2.98 | 8.37 | 5.21 | 12.14 | 5.29 | 12.14 | 5.29 |
| Pelvic torsion [°] | THA | -0.50 | 1.37 | -0.89 | 1.91 | -1.87 | 2.18 | -3.83 | 6.79 | -3.43 | 2.69 |
|  | Control | -0.47 | 1.12 | -0.92 | 2.19 | -1.34 | 1.83 | -2.53 | 2.73 | -4.22 | 3.11 |
| Surface rotation [°] | THA | 0.13 | 1.44 | 0.67 | 1.83 | 1.41 | 2.03 | 1.90 | 2.43 | 2.97 | 2.86 |
|  | Control | -0.05 | 1.29 | 0.24 | 1.97 | 0.51 | 1.82 | 0.88 | 2.50 | 2.02 | 3.36 |
| Lateral deviation [mm] | THA | -0.06 | 1.26 | 1.45 | 2.97 | 1.97 | 2.72 | 3.00 | 3.66 | 5.26 | 5.57 |
|  | Control | 0.20 | 1.80 | -0.11 | 3.30 | 0.77 | 2.83 | 0.96 | 4.97 | 4.14 | 7.11 |

| THA right side and control group. LLI simulation on the left side | | | | | | | | | | | |
| --- | --- | --- | --- | --- | --- | --- | --- | --- | --- | --- | --- |
| Parameter | Group | 5mm | | 10mm | | 15mm | | 20mm | | 30mm | |
|  |  | Mean | SD | Mean | SD | Mean | SD | Mean | SD | Mean | SD |
| Pelvic obliquity [mm] | THA | 2.58 | 1.69 | 7.01 | 3.86 | 7.01 | 3.86 | 9.92 | 3.51 | 13.16 | 4.46 |
|  | Control | 2.37 | 2.61 | 6.58 | 2.43 | 6.58 | 2.43 | 8.77 | 3.54 | 12.30 | 4.52 |
| Pelvic torsion [°] | THA | -0.79 | 1.09 | -1.97 | 1.83 | -1.97 | 1.83 | -2.86 | 2.17 | -4.04 | 3.30 |
|  | Control | -0.34 | 1.47 | -1.05 | 1.69 | -1.05 | 1.69 | -2.07 | 3.83 | -3.39 | 3.00 |
| Surface rotation [°] | THA | -0.17 | 1.57 | -0.53 | 2.02 | -0.53 | 2.02 | -1.41 | 2.92 | -3.10 | 3.78 |
|  | Control | 0.15 | 1.22 | -0.32 | 1.34 | -0.32 | 1.34 | -1.20 | 2.36 | -2.03 | 3.03 |
| Lateral deviation [mm] | THA | -0.34 | 1.83 | -2.06 | 2.75 | -2.06 | 2.75 | -2.93 | 3.90 | -6.09 | 5.86 |
|  | Control | -0.21 | 1.66 | -1.24 | 2.31 | -1.24 | 2.31 | -3.53 | 4.10 | -6.14 | 4.52 |
| THA left side and control group. LLI simulation on the left side | | | | | | | | | | | |
| Parameter | Group | 5mm | | 10mm | | 15mm | | 20mm | | 30mm | |
|  |  | Mean | SD | Mean | SD | Mean | SD | Mean | SD | Mean | SD |
| Pelvic obliquity [mm] | THA | 2.47 | 2.26 | 5.07 | 2.95 | 7.60 | 4.00 | 10.46 | 4.62 | 14.62 | 6.07 |
|  | Control | 2.37 | 2.61 | 4.03 | 3.15 | 6.58 | 2.43 | 8.77 | 3.54 | 12.30 | 4.52 |
| Pelvic torsion [°] | THA | -0.47 | 1.17 | -1.36 | 1.74 | -2.08 | 2.18 | -2.79 | 2.21 | -3.73 | 3.40 |
|  | Control | -0.34 | 1.47 | -0.43 | 2.03 | -1.05 | 1.69 | -2.07 | 3.83 | -3.39 | 3.00 |
| Surface rotation [°] | THA | -0.43 | 1.19 | -0.47 | 1.88 | -1.03 | 2.40 | -1.34 | 2.56 | -3.50 | 3.21 |
|  | Control | 0.15 | 1.22 | -0.30 | 1.77 | -0.32 | 1.34 | -1.20 | 2.36 | -2.03 | 3.03 |
| Lateral deviation [mm] | THA | -0.63 | 1.81 | -1.83 | 2.87 | -2.71 | 3.10 | -4.25 | 3.71 | -7.54 | 5.04 |
|  | Control | -0.21 | 1.66 | -0.67 | 3.21 | -1.24 | 2.31 | -3.53 | 4.10 | -6.14 | 4.52 |
| THA left side and control group. LLI simulation on the right side | | | | | | | | | | | |
| Parameter | Group | 5mm | | 10mm | | 15mm | | 20mm | | 30mm | |
|  |  | Mean | SD | Mean | SD | Mean | SD | Mean | SD | Mean | SD |
| Pelvic obliquity [mm] | THA | 2,49 | 1,58 | 2,49 | 1,58 | 10,44 | 2,57 | 10,44 | 2,57 | 14,85 | 4,03 |
|  | Control | 2,25 | 2,02 | 2,25 | 2,02 | 8,37 | 5,21 | 8,37 | 5,21 | 12,14 | 5,29 |
| Pelvic torsion [°] | THA | -0,43 | 1,17 | -0,43 | 1,17 | -2,21 | 2,76 | -2,21 | 2,76 | -3,36 | 3,54 |
|  | Control | -0,47 | 1,12 | -0,47 | 1,12 | -2,53 | 2,73 | -2,53 | 2,73 | -4,22 | 3,11 |
| Surface rotation [°] | THA | 0,18 | 1,64 | 0,18 | 1,64 | 0,85 | 2,18 | 0,85 | 2,18 | 1,79 | 2,72 |
|  | Control | -0,05 | 1,29 | -0,05 | 1,29 | 0,88 | 2,50 | 0,88 | 2,50 | 2,02 | 3,36 |
| Lateral deviation [mm] | THA | -0,49 | 1,86 | -0,49 | 1,86 | 0,87 | 3,37 | 0,87 | 3,37 | 3,06 | 5,53 |
|  | Control | 0,20 | 1,80 | 0,20 | 1,80 | 0,96 | 4,97 | 0,96 | 4,97 | 4,14 | 7,11 |
